# Training Comprehensive School Mental Health Providers: Reducing Shortages in Rural and High Needs Schools

**DOI:** 10.3390/bs16050648

**Published:** 2026-04-26

**Authors:** Erika Franta, Nicole R. Skaar, Megan Morse, Kerri Clopton, Stephanie Schmitz, David VanHorn

**Affiliations:** 1 Department of Psychology, Munroe-Meyer Institute, University of Nebraska Medical Center, Omaha, NE 68198, USA; megan.morse@unmc.edu; 2 Department of Learning, Leadership & Community, College of Education, University of Northern Iowa, Cedar Falls, IA 50614, USA; nicole.skaar@uni.edu (N.R.S.); kerri.clopton@uni.edu (K.C.); stephanie.schmitz@uni.edu (S.S.); david.vanhorn@uni.edu (D.V.)

**Keywords:** school psychology, school-based mental health, workforce development, Grow Your Own (GYO), dual-credentialing

## Abstract

This study addresses national shortages in school-based mental health (SBMH) providers, particularly in rural and high-needs areas, by examining two innovative training models designed to expand the school psychology workforce. The Grow Your Own (GYO) program respecializes practicing educators in rural communities to become school psychologists, while the Dual-Credentialing Clinical Training (DCT) model integrates school psychology training with supervised clinical experiences, leading toward educational certification and state mental health licensure. Program evaluation data were used to assess early implementation, feasibility, and success of both programs. In the GYO program, nine educators completed training, with eight employed in rural schools one to two years post-graduation, and average supervisor ratings meeting or exceeding the program’s competency expectations across all ten domains. In the DCT program, five trainees completed internship, four earned provisional mental health licenses, two progressed to independent licensure, and four became certified school psychologists. Together, findings indicate that place-based respecialization can strengthen rural retention, while dual-credentialing can expand clinical capacity and funding flexibility, creating complementary training models to help grow the SBMH workforce. Continued scaling and evaluation may enhance access to comprehensive SBMH services for students in under-resourced settings.

## 1. Introduction

Schools have become one of the primary, and often only, points of access to mental and behavioral health services for children and adolescents in the United States, particularly for students living in rural and high-needs areas (Atkins et al., 2017; National Association of School Psychologists [NASP], 2021). For many youth, school-based mental health (SBMH) providers are the first professionals to identify emotional or behavioral concerns, deliver prevention and intervention services, and connect students and families to additional supports (Weist et al., 2019). However, the capacity of schools to fulfill this role is increasingly constrained by persistent shortages of qualified SBMH professionals, including school psychologists, school counselors, and school social workers (National Association of School Psychologists [NASP], 2023; National Center for Education Statistics [NCES], 2024). These shortages are most acute in high-needs and rural settings, where difficulties recruiting and retaining personnel intersect with elevated levels of student need, limited access to community-based services, and geographic isolation (Atkins et al., 2017; Clopton & Knesting, 2006). As a result, schools are often tasked with responding to complex mental health concerns without adequate staffing, underscoring the urgency of developing training models that expand and sustain the SBMH workforce (National Association of School Psychologists [NASP], 2021).

The need for a robust school-based workforce is magnified by the severity and rising prevalence of mental health concerns among children and adolescents. Approximately 17% of youth ages 5–18 and 20% of adolescents ages 12–17 in the United States experience at least one diagnosable mental health disorder (Whitney & Peterson, 2019; Sappenfield et al., 2023). In 2023, suicide was the second leading cause of death among youth ages 10–14 and 15–24 (American Foundation for Suicide Prevention [AFSP], 2025; Centers for Disease Control and Prevention [CDC] & National Center for Health Statistics, 2025). From 2016 to 2023, the prevalence of mental illness among U.S. adolescents increased from 15% to 20.3% (Sappenfield et al., 2023), with additional increases linked to the COVID-19 pandemic (Campion et al., 2020; Hawes et al., 2022). Because schools are uniquely positioned to deliver early identification, prevention, and intervention services at scale—but can only do so when adequately staffed—addressing workforce shortages through innovative training and credentialing pathways is a critical component of strengthening comprehensive school mental health systems.

### 1.1. Shortage of School-Based Mental Health Providers

Recent data from the National Center for Education Statistics (NCES) suggested that less than half of public schools can provide effective mental health services to all students who need them. This is an approximately 10 percent decrease from five years ago as schools emerged from the COVID-19 lockdowns. In the same survey, public schools identified the most common barrier to providing adequate and effective mental health services as mental health provider shortages, inadequate funding, and limited access to highly qualified and licensed mental health professionals (National Center for Education Statistics [NCES], 2024).

National professional organizations have consistently documented these shortages: forty-eight states exceed the American School Counselor Association’s (ASCA) recommended student-to-school-counselor ratio of 250:1 (American School Counselor Association [ASCA], 2022), and the national average student-to-school-psychologist ratio is approximately 1065:1—more than double the National Association of School Psychologists’ (National Association of School Psychologists [NASP], 2023) recommended ratio of 500:1. These disparities represent a significant barrier to students’ access to timely, preventive, and comprehensive mental and behavioral health supports.

Inadequate staffing contributes to unmanageable caseloads, reduced capacity for early intervention, and limited implementation of evidence-based practices (Forman et al., 2021; Kelly et al., 2020). Students in understaffed schools are less likely to receive mental health services, more likely to experience unmet behavioral health needs, and at increased risk for academic difficulties, chronic absenteeism, and exclusionary discipline (Hoover et al., 2019). Workforce shortages are especially pronounced in high-need urban districts and geographically isolated rural communities, where recruitment and retention challenges intersect with elevated rates of childhood adversity, poverty, and trauma exposure (Atkins et al., 2017). These inequities underscore the urgency of developing innovative, scalable strategies to expand the SBMH workforce, particularly in these regions.

This manuscript follows federal definitions used by the U.S. Department of Education when referring to high-needs schools and high-needs areas. Under the Elementary and Secondary Education Act (ESEA), a high-need local educational agency (LEA) is defined as one that serves large proportions of students from low-income families, experiences persistent difficulty recruiting and retaining qualified personnel, and/or meets the federal definition of a rural LEA (U.S. Department of Education, 2018). Thus, rural status is explicitly included within the federal high-need designation rather than treated as a separate or parallel construct. While it is theoretically possible for individual high-needs schools to exist outside formally designated high-needs or rural LEAs, the schools participating in the training programs described in this study are all located within federally defined high-needs geographic areas, the majority of which are rural. Because the unit of analysis in this study is the school setting operating within these broader geographic and systemic contexts, the terms high-needs schools, high-needs areas, and rural areas are used interchangeably to reflect their overlapping classification under federal guidance and their shared workforce challenges related to recruitment, retention, and access to comprehensive school-based mental health services.

### 1.2. Barriers to Increasing School-Based Mental Health Providers

Efforts to expand the SBMH workforce are limited by how training and supervision requirements are defined across education and health systems. School psychologists are typically credentialed for school-based practice through state departments of education and educator licensure systems, which define the preparation requirements needed to serve in schools. Such licensing is not typically accepted by insurance agencies for reimbursement, making the funding of many school psychologists more difficult. In contrast, licensed mental and behavioral health providers are typically regulated through state health and human services agencies and boards, which define graduate training and supervised practice requirements for independent clinical practice. Nationally, most master’s-level counseling licenses require a graduate degree with supervised clinical training followed by a post-graduate supervised practice period, with states on average requiring 2000 to 3000 h of supervised experience before full independent licensure. Doctoral level school psychologists can also obtain this level of licensure without additional coursework, but Ed.S. level school psychologists often require additional coursework depending on the state requirements. Mental and behavioral health licensure pathways differ from education credentialing pathways and may create bottlenecks for training seeking to create a school mental health workforce to serve across both sectors to meet the complex needs of youth in schools.

The cost of higher education in the U.S. is rising, and from 2008 to 2023, average in-state tuition at public universities increased by $1060 for undergraduate students and by $1211 for graduate students, after adjustment for inflation. Financial issues are one of the common barriers to higher education for adult learners (e.g., Osam et al., 2017; Sarver & Miller, 2015). Examples of financial incentives for recruiting teachers include loan forgiveness, salary bonuses, tuition reimbursement (Feng & Sass, 2018), and grants (U.S. Department of Education, 2018). A review of programs providing loan forgiveness and/or service scholarship indicates these programs can be effective in recruiting and retaining educators when the financial support offsets the cost of education (Podolsky & Kini, 2016).

National workforce surveys conducted by NASP over the past two consistently show that the supply of school psychologists is insufficient to meet demand, with shortages most pronounced in rural, high-poverty, and linguistically diverse regions (Castillo et al., 2014; Curtis et al., 2002). Recruitment into graduate training programs remains impacted by limited program capacity, financial barriers, and a general lack of awareness of school psychology as a career among undergraduates (Bocanegra et al., 2019; Proctor & Romano, 2016). These barriers disproportionately affect candidates from racially and linguistically diverse backgrounds, contributing to a workforce that does not reflect the demographics of the students it serves, particularly in rural and high-needs areas (A. Goforth et al., 2021).

Retention challenges further exacerbate the workforce crisis. Barriers to retention such as high caseloads, role ambiguity, administrative pressures, and limited opportunities for professional growth as key contributors to burnout and attrition within the school psychologist workforce (Boccio et al., 2016; Walcott & Hyson, 2018). Early-career professionals are particularly vulnerable to retention challenges, with recent research indicating that inadequate supervision, insufficient induction support, and overwhelming service demands contribute to early exit from the field (Schilling et al., 2023). These retention issues are especially acute in rural and high-need districts, where working conditions are often more challenging and the workforce shortages are more persistent (Atkins et al., 2017). Collectively, these recruitment and retention barriers create a cycle of chronic understaffing that undermines schools’ ability to provide comprehensive mental health services.

### 1.3. Training Models

Two promising evidence-informed models exist that expand access to training and create new pathways into the school-based mental health profession in rural and high-needs areas. The first strategy is a Grow Your Own (GYO) respecialization program. The second model involves expanded clinical training pathways that allow candidates to obtain dual credentials, qualifying them both as certified school psychologists and as licensed mental health providers through state Departments of Health and Human Services (DHHS).

The two programs described in this paper emerged independently within statewide public institutions but were developed in response to these common challenges. Each institution leveraged its role within the state’s education and mental health infrastructure, along with longstanding partnerships with local education agencies and school districts, to design training models that address gaps in school-based mental health capacity (Espinoza et al., 2018; Frey et al., 2022). While the Grow Your Own (GYO) program emphasizes place-based respecialization and geographic retention in rural school districts, the Dual-Credentialing Clinical Training (DCT) program emphasizes scope-based expansion of services through integrated educational and clinical training (Guha et al., 2016; Frey et al., 2022). Together, these models illustrate complementary institutional responses to shared workforce shortages across Iowa and Nebraska and demonstrate how state-anchored programs can collectively contribute to strengthening school-based mental health services in high-needs schools (National Association of School Psychologists [NASP], 2021; U.S. Department of Education, 2025).

Iowa and Nebraska share similar educational and mental and behavioral health landscapes that provide important context for the development of the two training models described in this manuscript. Both states are characterized by large rural geographic regions, relatively small population centers, and school systems that rely heavily on regional service models to support students with specialized needs (Clopton & Knesting, 2006; National Center for Education Statistics [NCES], 2024). In both states, schools serve as a primary access point for mental and behavioral health services for children and adolescents, particularly in rural and high-needs communities where community-based providers are scarce (Atkins et al., 2017; National Association of School Psychologists [NASP], 2021). At the same time, Iowa and Nebraska experience comparable shortages in the school psychology workforce, with student-to-school-psychologist ratios that exceed national recommendations and recruitment and retention challenges that are especially pronounced outside urban centers (Affrunti, 2025; National Association of School Psychologists [NASP], 2023). These shared conditions have created parallel pressures on educator preparation programs and state institutions to develop training pathways that align with local workforce needs while remaining consistent with national professional standards (National Association of School Psychologists [NASP], 2021; U.S. Department of Education, 2025).

Across both training models, practitioner preparation is grounded in competency-based, applied training methods designed to develop the psychological, behavioral, and professional skills required for effective school-based mental health practice. Consistent with the NASP Practice Model (National Association of School Psychologists [NASP], 2020b, 2020d) and graduate training standards, trainees engage in sequenced coursework paired with progressively intensive supervised field experiences that integrate assessment, intervention, consultation, and systems-level problem solving within authentic school contexts. Training emphasizes active skill development through case-based learning, coached practice, performance-based evaluation, and iterative feedback from university faculty and field supervisors. Both programs prioritize combined didactic instruction with experiential learning mechanisms, such as supervised psychological assessment, delivery of evidence-based mental and behavioral health interventions, participation on school-based problem-solving teams, and consultation with educators and families, that promote the translation of knowledge into practice. Trainees develop core competencies including data-based decision making, intervention planning and implementation, ethical and culturally responsive practice, interdisciplinary collaboration, and professional identity as school-based mental health providers, thereby strengthening workforce readiness and capacity in high-needs school settings. Specifically, in the Grow Your Own model, these competencies are cultivated through place-based respecialization that embeds trainees’ psychological skill development within their existing school roles, while the dual-credentialing model emphasizes expanded clinical training, documentation, supervision, and treatment planning to prepare practitioners for intensive mental and behavioral health service delivery alongside school psychology practice.

Both training models described in this study are explicitly aligned with the National Association of School Psychologists’ (NASP) Domains of Practice and utilize competency-based training methods to prepare practitioners for school-based mental and behavioral health roles. Table 1 provides an illustrative mapping of selected NASP Domains to key training activities used in each model, highlighting how both programs operationalize competency development through distinct but complementary mechanisms.

Together, GYO respecialization pathways and DCT models offer scalable solutions to the national SBMH workforce crisis, including in rural and high needs areas. These approaches expand access to training, diversify the professional pipeline, and enhance the capacity of schools to deliver high-quality mental and behavioral health services. This paper presents two innovative training models designed to address the persistent shortage of school psychologists in rural and high-need schools, demonstrating strategic credentialing pathways to strengthen the workforce and improve student outcomes. The purpose of this paper is to describe the two programs and present early program evaluation data assessing implementation, feasibility, and initial success.

## 2. Program 1: Grow Your Own Rural School Psychologists

The first model focuses on transitioning existing educators, teachers, school staff, and administrators, into SBMH roles within rural high-needs schools. Designed for professionals already familiar with the academic system who are living and working in rural communities, the program equips them with the necessary graduate training to become licensed school psychologists. Through rigorous coursework, mentorship, and practical training alongside veteran school psychologists, educators develop expertise in psychological assessments, behavioral interventions, psychological consultation approaches, and student support strategies. Students in this grow your own (GYO) program commit to serving their local communities for at least three years post-graduation, fostering long-term retention and sustainability in rural mental health services.

### 2.1. Program Rationale

NASP recommends a school psychologist to student ratio of 1:500 to provide services across all 10 domains of practice. The most recent data suggest school psychologist shortages in almost every state across the country with an average of 1 school psychologist for every 1065 students (Affrunti, 2025). Rural areas are the hardest hit by shortages (Clopton & Knesting, 2006), and in the Midwestern state served by this program, the ratio is as high as 1:8000. While there is a growing interest in developing graduate programs that specifically address school psychologist shortages, the literature on effective programming is small and emerging. The literature on addressing teacher shortages is, at this time, more robust and provides key ideas to developing this school psychology GYO program.

Programs aimed to reduce teacher shortages, called teacher residencies, were designed to assist with the recruitment and retention of teachers in high need areas. Residency programs integrate theory and practice and provide more extensive field experiences than typical teacher education programs. Guha et al. (2016) reviewed 11 teacher residency programs and found 80 to 90% of program graduates stayed in the district for at least three years, while 70 to 80% were still in the district after five years. They suggested several key program components that resulted in graduate retention and program success. First, programs were built on strong university-district partnerships that aimed to recruit high ability individuals to fill specific needs. Guha et al. suggested that successful programs included lengthy field experiences and coursework that connected to the field experiences. Further, successful residency programs included high quality mentoring during the residency in addition to support following graduation. Another key component focused on the importance of having a cohort model to structure internal support for residents and the importance of high-quality placements where residents observed and interacted with experienced teachers. Finally, successful programs offered meaningful financial incentives.

Another study of teacher residencies also indicated success for programs of this type. The South Carolina Centers for the Re-Education and Advancement of Teachers in Special Education and Related Services Professionals provided financial support for individuals pursuing licensure as a special education teacher or related service provider (e.g., speech language pathologists, occupational therapists; Sutton et al., 2021). The report for the 2019–2020 year indicated 99% of program completers passed the exams needed for their new license, and 90% of program completers were employed in the same district the year following program completion.

NASP has long advocated for efforts to reduce school psychologist shortages (Vaillancourt Strobach & Oyen, 2021) and has suggested that respecialization and retraining of existing educators might be an effective solution (National Association of School Psychologists [NASP], 2021). NASP suggests that training programs should utilize online and distance education and evening and weekend classes to ensure that graduate training is more accessible to both working professionals and learners who live and work in areas distant from institutions of higher learning. This aligns with the idea of place-based education initiatives that center educator training in the community providing opportunities to explore and address community problems (Sobel, 2014; Powers, 2004). Further, NASP encourages local education agencies to offer incentives for employees to complete graduate training in school psychology, essentially growing their own school psychologists.

Grow your own programs are aimed at recruiting school employees, individuals wanting to change careers, and other community members into education programs (Espinoza et al., 2018). Over two-thirds of states have “grow your own” programs for paraeducators (Garcia, 2020). Schmitz et al. (2021) describes a grow your own school psychology program, and Bates et al. (2024) describes a similar program for school social workers. Both of these programs are partnerships between local education agencies and university graduate programs that recruit current educators into new careers as school mental health providers. In both programs, students take courses part time while continuing in their current educator role and receive substantial tuition support to offset the financial burden of returning to graduate school. Bates et al. reported that they had retained 100% of their students after one year in the two-year program and that students described positive perceptions of the program. Schmitz et al. reported 100% retention of their first-year students one semester into a two-and-a-half-year program, and as with Bates et al., students in the school psychology GYO program reported positive perceptions of the program. In both studies, students also reported barriers with both cohorts describing the intensity of the programs having a negative impact on their work–life balance. While it may be difficult to eliminate the stress of returning to graduate training while also working full time and attending to personal and family needs, graduate programs that aim to serve this population should do their best to reduce intensity without sacrificing rigor.

### 2.2. Recruitment Strategy

The goal of the program is to annually recruit five to six educators who are living and working in rural schools into the program. Guha et al. (2016) suggested that successful residency programs develop strong partnerships between the university and local education agencies. The University of Northern Iowa partnered with five different local education agencies (LEA) for this GYO program. All LEA partners were regional agencies that hire school psychologists and place them in area school districts and are interested in growing their school psychologist workforce in their rural communities. Two of the LEA partners serve regions of the state that are entirely rural, and the other three agencies each have one urban center with most of the agency serving rural communities. Leaders of these area education agencies (AEAs) reported that it is difficult to recruit school psychologists to work in rural schools, and the aim of this program to train educators living and working in their rural communities was appealing as the ratios of school psychologists to students in the rural districts ranged from 1:1600 to 1:4000, up to 8 times the recommended ratio. These partnerships have remained strong since 2019. Recently, these five partner AEAs, along with two additional AEAs, collaborated to extend this GYO program further.

Guha et al. (2016) also suggested that successful programs should tailor recruiting efforts to bring in high ability professionals. The AEA partners recruit applicants from their existing employees, their service area school districts, and from related service agencies in the area (e.g., vocational rehabilitation). They attempt to recruit educators who have demonstrated the potential to successfully complete a rigorous graduate program and excel as employed school psychologists. Over the past three years, the average number of applications has been 12, and faculty have been able to be selective with admissions. The admissions application includes reviewing transcripts, recommendation letters, and a personal statement from the applicant. Faculty complete a rigorous review of each applicant’s graduate transcript to ensure that courses being transferred from their master’s programs meet the requirements of the courses residential program students take. Faculty also consider work experience as they ensure applicants have the skills needed for successful completion of the program. Within the five cohorts accepted into the program from 2019–2024, most have been special education coaches (experienced special education teachers who support special education classroom teachers through consultation and assessment of students with disabilities), one was a school leader, one was a systems coach, one was a school counselor, one was a speech and language pathologist, one was a social worker, and four were general or special education teachers.

### 2.3. Training Components

The key components suggested by Guha et al. (2016) and NASP’s recommendations were consulted for the development and implementation of this GYO program. The program aims to recruit and retrain rural master’s level educators as school psychologists to reduce the shortage of school psychologists and increase the school mental health workforce in rural communities. GYO students earn the Ed.S. degree after transferring 16 master’s level credits and taking 44 credits of coursework and internship at the university. Graduates are eligible for licensure by the state department of education and can choose to obtain their Nationally Certified School Psychologist credential. The program was developed in 2019 and funded by the US Department of Education as part of the Mental Health Service Provider (MHSP) grant program. This program was extended through the same MHSP grant program in 2023. GYO students are accepted into the program in cohorts of five to six students. Students in the cohorts take all their courses together throughout the program. Benefits of the cohort model include students developing a sense of community and support through a stressful and rigorous graduate program, students developing both professional and personal relationships with their cohort members, and increased student engagement and program satisfaction (Martin et al., 2017; Mauldin et al., 2022).

The foundation of the program coursework and field experiences is the NASP graduate training standards (National Association of School Psychologists [NASP], 2020b). The NASP standards require students to have courses with assignments that measure the 10 NASP practice domains: Data-based decision making; Consultation and collaboration; Academic interventions and supports; Mental and behavior health services; School-wide practices to promote learning; Services to promote safe and supportive schools; Family, school and community collaboration; Equitable practices for diverse populations; Research and evidence-based practice; and Legal, ethical, and professional practice. The program of study (Table 2) includes courses and field experiences students take during the program and courses for which students will transfer credit from their previous master’s level coursework.

Most courses are provided through synchronous online instruction; however, the assessment courses (see below MEASRES 6283, MEASRES 6282, MEASRES 6284, and MEASRES 6287) have a face to face component. This was intentionally planned to address the need for students to build relationships with each other and with program faculty and for students to get in-person instruction and practice with assessment materials. The in-person instruction is achieved in two ways. During the academic year, the instructor travels to student locations to provide in-person learning. The instructor rotates the in-person instruction to different areas of the state with a goal of offering in-person instruction to all students in the program multiple times. Students who cannot travel to the in-person session attend via Zoom. The other method of offering in-person instruction is through summer residency on the university campus. Students come to campus two weeks each summer for in-person assessment instruction.

Students complete 360 h of practicum during the second academic year of the program. Practicum experiences are typically done in a rural school and must be supervised by a practicing school psychologist. Completing practicum experiences in rural areas is intentional and aligns with place-based education where learning is centered in the community in which future educators will practice (Sobel, 2014). This has been the most difficult aspect of the program because students are working full time during the school day. Further difficulty comes from the shortage of school psychologists in rural schools. At times, our practicum students must travel up to an hour for supervision with a school psychologist. To support practicum hours, the program requires students to either take a position with a partner AEA during the year they are on practicum or to get a signed document from their employer stating that they are able to attend practicum one or two days per week during their second year in the program. This arrangement has been in place for the most recent three years of the program, and 82% of students either already work at the AEA or transition into a position with the AEA during the practicum year. This option has created an alternate employment opportunity in situations where a classroom teacher’s employer may not be accepting of them missing work one to two days a week throughout the school year. However, two students continued to work for local education districts throughout the program with the support of their employer.

The final year of the program is a full-time, 1500-h internship with a partner AEA and are assigned to serve rural school districts. AEAs consider school psychology interns as first year school psychologists and provide them a full first year salary and benefits. Because the interns have years of experience working in schools, they can often receive higher salaries than other first year school psychologists because they are credited for their years of K-12 experience on the salary schedule.

### 2.4. Supervision Structure

Guha et al. (2016) identified that successful teacher residency programs include high quality mentoring during the residency in addition to support following graduation. The GYO program is led by four faculty with one of the faculty members assigned specifically to the GYO program. This person teaches many of the courses in the program, and travels across the state to meet students in person during the assessment courses. This is the person who interacts with GYO students regularly, and he works with the agencies that employ GYO students to ensure they are allowed the time they need to attend courses and practicum. This dedicated program coordinator is vital to the success of the program. This person’s sole responsibility is the learning and support of the GYO students without any responsibilities to the residential school psychology program or the obligations of university service and scholarship as with the tenured faculty in the program.

During internship, students in the GYO participate in both field and university supervision with the residential program students. Interns must obtain at least two hours of supervision with a veteran school psychologist. Each intern is assigned a school psychologist supervisor within their agency. Given the rural settings in which the GYO interns work, supervision can be a challenge. Supervisors and interns often drive to a central location to meet or take turns traveling the distance to one another’s schools. While NASP guidelines (National Association of School Psychologists [NASP], 2020b) require supervision to be face to face, the pandemic required supervision to move online. Since then, NASP has recognized online, synchronous supervision as acceptable. While field supervision is strongly encouraged to be face to face when possible, university supervision occurs solely through online, synchronous methods. GYO students are spread across the state in rural schools, and the university is located in a small city in the northeastern quadrant of the state. University supervision is done by an adjunct faculty member who is a licensed, doctoral level school psychologist leader working at a partner agency. The program coordinator also supports university supervision. Together, they ensure all interns are supported through monthly scheduled check-ins and are accessible when interns have questions and concerns.

### 2.5. Funding Model

Finally, all GYO students are provided with financial support throughout the program. Both National Association of School Psychologists (National Association of School Psychologists [NASP], 2021) and Guha et al. (2016) suggest providing financial incentives because the financial burden of going back to graduate school can be a barrier for retraining. Schmitz et al. (2021) shared that students in the GYO program reported they would not be able to attend the program without the financial support. Students receive an 85% reduction in tuition and fees. They also receive funds to pay for their books, travel to campus in the summer, travel to instructional sessions during the academic year, their internship license, and their PRAXIS exam, which is required for graduation from the program. Students also receive free PREPaRE training and receive financial assistance to attend regional conferences to supplement their learning. The cost of completing the program is approximately $4000 for 44 credits of graduate coursework and field experiences.

### 2.6. Evaluation Methods

#### 2.6.1. Participants

Graduates of the program are nine students who, prior to starting the program, all lived and worked in rural schools and had obtained a master’s degree in education or a related field. They received master’s degrees in special education, social work, school counseling, educational leadership, and early childhood education, and they had 2–30 (mean = 14.9) years of experience working in educational settings prior to starting the GYO program. All graduates are female and White, which is representative of the educational professionals in rural areas of the state (Iowa Department of Education, 2024). Since the program aims to recruit educational professionals living and working in rural areas of Iowa, the opportunities to recruit a more diverse cohort of graduate students for this program is limited.

#### 2.6.2. Procedures

Data collection was completed as part of the evaluation of the program, and it was used for research with the approval of the university Institutional Review Board (IRB). All participants completed an online consent form asking permission to use their program data for research purposes. To determine effectiveness for this study, final field supervisor internship evaluation data were gathered and analyzed. The internship evaluation completed by all internship supervisors includes several items across each of the 10 NASP practice domains (see Table 3) rated on a Likert-type scale of 1 (Not Satisfactory) to 10 (Exceptional). Internship supervisors completed intern evaluations blind to use of the ratings beyond evaluation of the individual students. An average NASP domain rating is calculated by averaging the ratings of items across each domain. Only one rater completes the internship evaluation; therefore, no inter-rater reliability data are available for this measure. In addition to internship evaluation data, current employment information was collected from publicly available employer websites that are kept up to date with current employee information.

### 2.7. Outcomes

One goal of the GYO program is to retain school psychologists in rural school settings. Of the nine graduates of the GYO program, eight (89% of total number of graduates) continue to work in rural school districts 1 to 2 years post-graduation. Not all graduates have remained in their original rural school psychologist positions since graduation, but they continue to serve rural schools. One graduate now works in a rural school district in a neighboring state, one took a position in a rural area of Alaska, and another graduate recently took a position supporting therapeutic schools in a rural school district. To compare with a traditional residential school psychology program, employment data were gathered for students who graduated from the residential school psychology program at the same university. Employment data is publicly available on local education agency websites. Approximately 43% (10 of 23 graduates) of students who graduated in the same time period as the GYO students have chosen and maintained a practice in a rural school district 1 to 2 years post-graduation.

Another goal of the program is that GYO students will meet the rigorous academic expectations of the program and achieve a minimum standard of success in the field. One measure of student success is their end of program evaluation from their internship supervisor. Internship supervisors are in a unique position to observe school psychological practice at the students’ school(s) and provide evaluation of expected skills. For the final internship evaluation occurring in May of the internship year, the program expectation is that interns receive an average domain rating of at least 6 (highly competent) from their field supervisor. Average ratings across all students for each of the 10 NASP domains of practice exceeded this expectation (see Table 3); however, not all students met this expectation in all NASP domains. One student received a rating of 5 (Competent) in Research and Evidence-Based Practice (Domain 9). Another student received scores nearing 6.0 in Equitable Practice for Diverse Student Populations (Domain 8) and in Research and Evidence-Based Practice (Domain 9). The highest average ratings across all students were in Legal, Ethical, and Professional Practice (Domain 10) and in Equitable Practices for All Students (Domain 8). The lowest average ratings across all students were in Research & Evidence-Based Practice (Domain 9) and Data-Based Decision Making (Domain 1).

## 3. Program 2: Dual-Credentialed School Psychologists in High Needs Settings

The second model focuses on the clinical training required for pre-degree certification and the post-degree supervised clinical training required for most state mental health licensure. Trainees engage in supervised fieldwork within high-needs schools in both rural and urban settings to work toward both school psychology certification and mental health licensure in the state, under the supervision of a school psychology-trained licensed psychologist or independent licensed mental health professional. Through hands-on experience, interns learn evidence-based intervention strategies and assessment skills; engage in clinical consultation with educators, administrators, and other school-based mental health staff; and tailor crisis response approaches to the unique challenges faced by students in these settings. By integrating these trainees into the school environment early in their professional development, the model ensures a steady pipeline of school-based mental health professionals prepared to address mental health needs in underserved communities. Funding for this training program was supported by the U.S. Department of Education, through Grant S184X230117 to the University of Nebraska Medical Center. The opinions expressed are those of the authors and do not represent views of the Institute or the U.S. Department of Education.

### 3.1. Program Rationale

Dual-credentialing clinical training programs for school-based mental health professionals are grounded in a theoretical orientation that deliberately bridges the education and mental and behavioral health systems in which school-based mental health professionals practice. These programs are grounded in implementation science and competency-based professional preparation frameworks, which emphasize the importance of aligning training context, supervision structures, and performance expectations to promote skill acquisition, generalization, and sustainability in applied settings (Forman et al., 2021; Frey et al., 2022). From an implementation science perspective, the DCT model incorporates core implementation supports, including readiness-building activities, coached and supervised practice, structured performance feedback, and ongoing fidelity monitoring, to facilitate the uptake and sustained delivery of evidence-based mental and behavioral health services within real-world school contexts (Forman et al., 2021). This theoretical grounding informed both program design and evaluation by intentionally structuring training experiences to support progression from closely supervised practice toward increasing professional autonomy, while assessing trainee competence using end-of-year evaluations aligned with NASP Domains of Practice and graduate program benchmarks (National Association of School Psychologists [NASP], 2020b). Explicitly situating the DCT program within an implementation science framework clarifies the mechanisms through which dual-credentialing supports practitioner competence, role integration across education and mental health systems, and workforce capacity development, thereby enhancing the model’s generalizability to other high-needs school settings (Frey et al., 2022; Lever et al., 2014).

Successful dual-credentialing programs for school psychologists serving as school-based mental health professionals align with the National Association of School Psychologists (NASP) Practice Model’s ecological and problem-solving orientation involving data-based decision making, consultation, prevention/intervention, and mental/behavioral health services, while extending the scope into the mental and behavioral health practice that is often restricted without additional licensure. In this orientation, trainees are developed as hybrid professionals who can deliver evidence-based assessment and intervention within a multi-tiered system of supports and in alignment with federal and state special education law, while also meeting expectations of clinical mental health practice, such as treatment planning, documentation, supervision, and ethical/legal standards for licensed providers.

The Dual-Credentialing Clinical Training (DCT) program for school psychologists focuses on a dual-credentialing clinical training model outlined by Frey et al. (2022) by providing school-based mental health clinical training to school psychologists during their internship year to meet requirements by both (1) the state-department of education to become certified school psychologists in the state and (2) completing the requirements to be eligible for a state-department of health and human services mental health licensure. Its theory of action translates this into practice by training future school-based mental health clinicians in behavioral principles to influence individual, home, school, and societal systems. Trainees use evidence-based practices implemented within multi-tiered systems of support (MTSS) to improve student functioning. Core competencies for the trainees align with both the National Association of School Psychologists and the American Psychological Association program accreditation guidance. Trainees build competencies across a wide range of areas, including intervention, consultation, evaluation, and cultural competence. School mental health specific competencies highlight interprofessional collaboration, support across academic and behavioral domains, and personal and professional development, as outlined by Lever et al. (2014).

Dual-credentialing models support the financial sustainability of school-based mental health systems by preparing practitioners to deliver licensure-eligible clinical services that can be supported through grants, district funding, or third-party reimbursement. By training school psychologists to meet licensure-related documentation and supervision standards, programs create pathways for clinical service delivery that extend beyond exclusively education-funded roles. This approach aligns with national policy trends emphasizing flexible credentialing and diversified funding streams as strategies to stabilize and expand the behavioral health workforce in schools (Frey et al., 2022; National Governors Association, 2024; U.S. Department of Education, 2025).

### 3.2. Recruitment Strategy

This model addresses recruitment and retention concerns by meeting the school-based mental health field’s growing need for cross-disciplinary expertise and flexible credential pathways (Frey et al., 2022; U.S. Department of Education, 2025). The model addresses shortages by strengthening the capacity of the workforce by preparing practitioners who can navigate federal and local educational and health law, while delivering clinically robust services to students with complex, co-occurring needs (National Governors Association, 2024). The DCT program utilizes established partnerships with two of the three universities in the state that offer the educational specialist degrees for school psychology to provide in-person or virtual presentations to their first-year students to overview the dual-credential training program. Of note, the department in which the DCT program resides, has a long-standing partnership with the third training program for various training opportunities and as such did not require additional meetings with the faculty and/or their students.

### 3.3. Training Components

Dual-credentialing clinical training programs aim to strengthen the school-based mental health workforce by creating intentional pipelines into high-need school settings. By recruiting school psychology trainees during their coursework, providing paid clinical training experiences, and supporting progression toward full mental health licensure, these models address workforce shortages that limit access to mental and behavioral health services. Programs that prepare school-based mental health practitioners who can navigate both educational and mental and behavioral health systems increases workforce flexibility, improves retention, and enhances capacity to serve students with complex and co-occurring mental, behavioral, and academic needs, particularly in underserved rural and urban communities (Frey et al., 2022; National Governors Association, 2024; U.S. Department of Education, 2025).

Dual-credentialing training programs anchor clinical training to the mental and behavioral health services with a multi-tiered system of supports (MTSS), allowing school-based care to remain integrated with educational frameworks while meeting mental and behavioral health clinical standards of care. DCT program trainees complete at least 1500 h during their first year of clinical training, typically referred to as their “internship year.” Then, trainees are quired to complete an additional 3000 h post-masters to fulfill full mental health licensure requirements, with at least half of those hours being direct clinical services.

In the DCT program, trainees are prepared to deliver services across universal, targeted, and intensive tiers, with particular emphasis on Tier 2 and Tier 3 interventions, that address mental and behavioral health needs of students. This alignment supports data-driven decision making, coordinated teaming, and progress monitoring, while ensuring that clinical services provided in schools meet expectations for effectiveness, accountability, and responsiveness to student need.

The DCT program prepares clinicians to work across all tiers of support, in alignment with the NASP practice model. Trainees are supervised in delivering universal or tier one support through engaging in universal mental health promotion practices within the district, delivering psychoeducation to educators within the building served, and supporting the building level problem solving teams. Trainees are supervised in delivering tier two practices through implementation of targeted group interventions and consultation with building-level coaches. Trainees are supervised in delivering tier three practices through delivering intensive individual/family therapy, psychoeducational evaluations, individual academic and/or behavioral interventions, and consultation on individual education plans or 504 plans. Effective School-Based Teaming Trainees practice aligning their clinical strategies with school-based MTSS and sharing treatment plans and progress monitoring data with school teams, fostering collaborative, data-informed care.

Successful family, school, and community partnerships is a cornerstone of the training program. Trainees are trained in offering flexible participation options in multi-tiered services, including telehealth and in-person consultations. Partnerships with schools and community providers are built on mutual curiosity, clear communication, and shared goals for student well-being. Trainees complete clinical placements across urban and rural districts, engage in team-based consultation, and collaborate directly with educators, caregivers, and community partners under the supervision of licensed providers. These experiences emphasize integration with existing school systems and responsiveness to local context. Further, trainees gain experience engaging with partners to support professional development and implementation of best practices in comprehensive school mental health systems.

Although there is no official coursework for the DCT program, all trainees of this program engage in an intensive, onboarding bootcamp prior to engaging in the school setting. This bootcamp involves didact learning on electronic health records, The National School Mental Health Best Practices and Implementation Guidance Modules (NCSMH & MHTTC, 2019), history and context of local school systems, federal and state special education law, and various clinical topics. All trainees participate in training to certification on the Autism Diagnostic and Observation System (ADOS) prior to beginning their clinical work. To illustrate how intensive onboarding activities complement didactic training throughout the internship year, Table 4 provides a timeline of the didactic schedule.

Trainees also engage in learning opportunities through our partner districts. Trainees are invited to attend Professional Learning Communities (PLCs) with other school psychologists in the district; Response-to-Intervention District-wide trainings focusing on assessment tools, interventions, how to use data-based decision making from different professional lenses; Functional Behavioral Assessment practices for the districts served, psychoeducational Evaluation Practices for ELL Students; Strengths-based Evaluation Practices.

### 3.4. Supervision Structure

A distinguishing feature of DCT programs is the integration of compliant supervision, documentation, and professional practice standards required for independent mental health licensure alongside school psychology certification within the training state. Trainees in the DCT program receive structured individual and group supervision from licensed clinicians qualified to oversee clinical practice, ensuring compliance with state licensure requirements while also meeting educational internship expectations. Clinical supervisors meet for two hours, minimum, with their trainees each week to discuss clinical documentation, ethical standards, case conceptualization, treatment planning, and progress toward competencies. Clinical Supervisors provide a mid-year and end-of-year evaluation of the trainees in alignment with their degree program’s requirements, as well.

### 3.5. Funding Model

Trainees are offered financial stipends for their training year. Funding aligns closely with stipends offered by local districts for similarly experienced school and mental health professionals. The training program also funds mileage to and from training sites, in accordance with university travel rules. Funding for this training was supported by the U.S. Department of Education, through Grant S184X230117 to the University of Nebraska Medical Center.

### 3.6. Evaluation Methods

#### 3.6.1. Participants

Since the Dual-Credential Training Program’s inception, five trainees have successfully completed the program. Four of the trainees were white females and one trainee was a white male. All were enrolled in a school psychology graduate training program, had completed two full years of coursework, and were engaging in their final year of internship training prior to graduation and credentialing upon enrollment into the program. Of the five trainees, three began their clinical internship with a master’s degree and were provisionally licensed mental health professionals through the department of health and human services.

#### 3.6.2. Procedures

Data collection was completed as part of the evaluation of the program and grant reporting for the U.S. Department of Education Grant S184X230117, and it was deemed exempt by the university Institutional Review Board (IRB). All credentialing data was gathered through self-report during entrance and exit interviews from the program. All competency data was gathered through end of year evaluations completed by the clinical supervisors during exit activities. Any additional current employment information was collected from publicly available websites and phone interviews.

Evaluation of trainee competency within the Dual-Credentialing Clinical Training (DCT) program relied on end-of-year internship evaluations completed by clinical supervisors using assessment instruments required by each trainee’s graduate training program. As trainees enrolled in the DCT program originate from different NASP-aligned school psychology graduate programs, the specific evaluation instruments, rating scales, and competency indicators varied across programs. Although all instruments were aligned with the National Association of School Psychologists’ (NASP) Domains of Practice, they were not identical in item content, scaling, or reporting format. In addition, access to full item-level evaluation data varied due to program-specific data sharing policies, and not all raw evaluation forms were available for secondary analysis by the DCT program.

Competency data for DCT trainees are reported at an aggregate level. Across all five trainees, end-of-year evaluations indicated that trainees met or exceeded the level of “competent” in all NASP Domains of Practice, as defined by their respective graduate training programs. All trainees also received ratings of “satisfactory” or higher on professionalism and ethical practice indicators. While these aggregated findings provide evidence that trainees achieved expected competency benchmarks for entry-level practice, they do not permit direct comparison across trainees or fine-grained analysis by domain. Future program evaluation efforts will prioritize standardized competency measurement and data-sharing agreements across partner training programs to support more detailed outcome reporting.

### 3.7. Outcomes

The main outcomes for the DCT program include growing dual-credentialed, competent school-based mental health providers to serve in high needs areas. Regarding credentialing of the DCT program trainees (Table 5), four of the five trainees ended their clinical training year with a provisional mental health license in the state. Of the five enrolled, three continued to work toward their provisional mental health licensure in their practice state and two have successfully achieved the required amount of supervised clinical hours to obtain their independent mental health license in their practice state. Of the five enrolled, four become certified school psychologists in their practice state or as nationally certified school psychologists.

Regarding placement of the DCT trainees (Table 6), four of the five trainees completed their one-year internship and then moved to another site to complete their training hours for mental health licensure in their practice state. One of the five trainees completed an additional two years at the site to complete their independent mental health licensure.

Another goal for the DCT program, is that trainees meet competence within the domains of clinical practice outlined by their academic training program, during their internship year. This is best indicated by their end of program evaluation from their clinical supervisor who oversees their practice in June of their internship year. Because trainees come from differing academic programs, the specific competency requirements vary, although all are aligned with the NASP Domains of Practice. All trainees in the DCT program received scores of “Meets Competence” or higher across all NASP Domains of Practice. Further, all five trainees received ratings of “Satisfactory” or above on professionalism domains as outlined by their program evaluation tools.

## 4. Discussion

This article describes two complementary training approaches developed in response to persistent shortages in the school-based mental health (SBMH) workforce. Both models were built to strengthen the pipeline of school psychologists, but they do so through different leverage points. One approach focuses on “place-based” preparation by respecializing current educators already living and working in rural communities, while the second approach focuses on “scope-based” preparation by training school psychology interns to deliver clinically robust services while progressing toward clinical mental health licensure. Together, these models align with broader national efforts to expand access to prevention and intervention supports in schools and respond to ongoing constraints in both school mental health and school psychology capacity (Frey et al., 2022; National Association of School Psychologists [NASP], 2021; U.S. Department of Education, 2025).

Recruitment and retention barriers in the school-based mental health (SBMH) workforce are well documented, particularly in rural and high-needs school settings where financial strain, limited access to qualified supervision, professional isolation, and high workload demands contribute to persistent staffing shortages (Atkins et al., 2017; Clopton & Knesting, 2006; National Association of School Psychologists [NASP], 2023). These barriers also intersect with issues of diversity and equity, as structural constraints within graduate training, supervision, and licensure pathways can disproportionately limit access for candidates from historically underrepresented backgrounds (Bocanegra et al., 2019; A. Goforth et al., 2021). In recognition of these intersecting challenges, the Grow Your Own (GYO) and Dual-Credentialing Clinical Training (DCT) programs were intentionally designed to address both workforce shortages and equity concerns through embedded, structural program features rather than relying solely on individual persistence or informal supports.

Financial strain is a barrier to both recruitment and retention and has been identified as a deterrent for candidates considering graduate training in school-based mental health professions, particularly those from economically marginalized backgrounds (Osam et al., 2017; Podolsky & Kini, 2016). Both programs directly address this barrier through substantial financial supports, including tuition reduction or coverage, paid internships or stipends, and reimbursement for licensure-related expenses. These supports reduce the financial burden associated with graduate education and supervised practice, enabling trainees to complete rigorous preparation while maintaining financial stability. Flexible scheduling and place-based training further reduce access barriers by allowing working professionals to remain employed in their local communities while completing training, an approach shown to support recruitment and retention in rural and high-needs contexts (Espinoza et al., 2018; Garcia, 2020).

Inadequate access to qualified supervision is another central contributor to early-career stress, burnout, and attrition within the school psychology and SBMH workforce, particularly in rural settings where supervisors may be scarce (Boccio et al., 2016; Clopton & Knesting, 2006). To address this barrier, both programs embed formalized and structured supervision models as core design features rather than ancillary supports. The GYO model leverages partnerships with regional education agencies to ensure consistent access to experienced school psychologist supervisors within trainees’ home regions, while the DCT model integrates supervision from licensed clinicians qualified to support both school-based practice and progression toward mental health licensure. Clear supervision expectations, defined communication channels, and structured accountability are intended to reduce role ambiguity and promote competency development during high-risk professional transition periods (Edwards & Sullivan, 2014; Schilling et al., 2023).

Recruitment strategies across both programs are also intentionally aligned with equity and workforce sustainability goals. Faculty engage in targeted outreach and program visibility efforts through presentations and tabling at national, state, and regional professional conferences, including the National Association of School Psychologists (NASP) and the Annual School Mental Health (ASMH) Conference, as well as state association and specialty group meetings. These activities are designed to increase awareness of training pathways among graduate students, early-career professionals, and practicing educators from diverse educational, racial, linguistic, and socioeconomic backgrounds, while also strengthening regional and national professional networks that support collaboration, funding development, and long-term program sustainability (Bocanegra et al., 2019; National Association of School Psychologists [NASP], 2021).

Finally, cohort-based training models and structured supervision across both programs are central mechanisms for addressing professional isolation and fostering belonging, which are particularly salient for trainees practicing in rural and high-needs schools and for individuals from underrepresented backgrounds (Martin et al., 2017; Mauldin et al., 2022). Training content explicitly emphasizes culturally responsive practice, ethical decision making, and equitable service delivery within school settings, aligning practitioner preparation with the demographic realities and systemic inequities experienced by students and families served in these contexts (A. N. Goforth et al., 2017; National Association of School Psychologists [NASP], 2020b). Collectively, these intentional design features illustrate how recruitment, retention, diversity, and equity challenges can be addressed through coordinated structural solutions, reinforcing the importance of competency-based, context-responsive training models for strengthening the SBMH workforce in high-needs school settings.

The GYO program was established to address the challenges that rural districts face, such as disproportionate recruitment and retention challenges that do not meet rural staffing needs. In these contexts, respecializing educators with strong school-system knowledge and ties to the local community is a practical strategy for stabilizing services over time (Clopton & Knesting, 2006). This is consistent with broader “grow your own” approaches that emphasize recruiting school employees and community members into professional roles, which may improve fit and retention because trainees are already embedded in local contexts and understand the realities of rural schooling (Espinoza et al., 2018; Garcia, 2020). The GYO school psychologist program intentionally recruits educators living and working in rural areas. This recruiting strategy is different from recruiting to a traditional residential program (Bocanegra et al., 2022), and these early results suggest this strategy may increase the number of school psychologists to remain working in rural settings over time.

The data described above suggest that the GYO program developed based on recommendations from Guha et al. (2016) was initially successful in recruiting, retraining, and retaining school psychologists for rural practice. Not all graduates have remained in the state or in their initial school psychology positions, but all but one of the graduates have continued to practice in rural settings. With so few school psychologists practicing in rural schools, this increase of eight school psychologists will increase access to school mental health services for an estimated 14,000 students. Further the program was successful in training the GYO students in all 10 domains of school psychological practice. While there was room for improvement in a couple areas for two individual students, the average scores for program students across all NASP domains were at or above the minimum criteria expected for the end of internship. When a student receives a rating below the expected minimum, faculty consider requiring remediation in that domain. Remediation may include additional internship hours, a book study, or case work that is then reviewed by the school psychology faculty.

The DCT program was developed in response to schools increasingly needing staff who can deliver clinically robust services within the school system, yet professional roles and credential requirements often differ across educational and behavioral health systems. By intentionally preparing trainees to function as hybrid professionals who can navigate school systems while progressing toward clinical mental health licensure, the DCT program aims to expand workforce flexibility and increase service capacity in high-need school settings. This approach also aligns with broader policy trends that emphasize layered behavioral health credential pathways to address workforce gaps and expand access to services for children and adolescents in community-based settings, including schools, in alignment with the recommendations put forth by the U.S. Department of Education (2025) and the National Governors Association (2024).

A strength of the DCT program is that the model integrates training expectations from school psychology and clinical mental and behavioral health practice by emphasizing supervision structures, documentation standards, and competency development that support licensure progression alongside school psychology certification. The model remains grounded in an ecological, problem-solving approach consistent with the NASP Practice Model, while further extending clinical preparation into mental and behavioral health regulated service delivery. This integration is reinforced through implementation-oriented training structures such as didactic readiness-building, coached practice, and role integration within school systems. The data described above suggest that the DCT program based on Frey et al. (2022) dual-competence model has been initially successful in establishing educationally credentialed and provisionally licensed school psychologists. With documented movement toward independent licensure for all trainees in the sample, these data suggest that the DCT program offers a pathway that serves as a viable pipeline into dual-competence roles. Not all graduates have remained in the state or in their initial school psychology positions, but all but one of the graduates have continued to work within the educational setting in high-needs or rural schools and populations.

### 4.1. Challenges and Lessons Learned

Both programs exemplify strong approaches to building the school-based mental health workforce through school psychologist training opportunities. High-impact elements of both models include robust partnerships, establishing strong and compliant supervision, offering financial support to trainees through stipends and travel coverage, and engaging in a cohort training structure. By grounding training in systemic, evidence-based frameworks and fostering strong school and community partnerships, the programs offer a replicable model for addressing mental health disparities among youth and alleviating professional shortages in high-need schools.

Taken together, these models illustrate complementary strategies for strengthening the SBMH workforce through school psychology training. The GYO program primarily strengthens geographic retention by developing professionals who are already rooted in rural communities, while the DCT program primarily strengthens clinical capacity and cross-system practice flexibility by preparing school psychologists for dual roles across educational and behavioral health expectations. Across both models, common “active ingredients” include strong partnerships, structured mentoring and supervision, financial supports, and workforce development strategies associated with successful efforts in high-need and rural settings.

The first cohort of students in both programs started their experiences during the 2019–2020 academic year as schools and universities across the country moved online. While courses for the GYO program were originally planned to be largely online and synchronous, there were planned face to face elements that were not able to occur that first year. While didactic training and supervision practices for the DCT program were originally planned to include some online opportunities, the clinical training was intended to be face-to-face and services had to be moved to a tele-behavioral health model. This was the first challenge of many faced by the faculty, students, and supervisors.

Other challenges across both programs, included recruiting students and trainees who are a good fit for rigorous study and training, difficulty with the logistics of practicum experiences, and preparing school psychologists specifically for practice in rural and high-needs areas. Through problem-solving these challenges, program faculty and supervisors have focused on flexibility, adherence to NASP’s rigorous graduate standards, and place-based educational experiences as strengths of the programs. Increased outreach is necessary to recruit the types of students that are both qualified and interested in training of this nature. Partnerships with local school districts and regional educational agencies are key in recruiting high quality students. Recruiting students for the GYO and DCT programs has been successful due to partner educational agencies reaching out to local educators and students who have shown interest in school-based mental health practice. The close relationships between rural partner agencies and rural educators enhances credibility and trust that the university may not have developed alone. Further, those who have graduated from the program are excellent spokespeople for the program and continue to successfully recruit the next cohorts of GYO and DCT program students. Again, alumni of the program have immediate credibility because they have completed the program and can provide information from the student perspective.

Supervision of practicum, internship, and clinical licensure experiences has presented challenges. The GYO program is tailored for working educators, which means that attending practicum during the school day is logistically difficult. The GYO program faculty are flexible with the types of experiences that can count for practicum hours; however, there are limits to this flexibility. GYO students must take part in school psychological work with the supervision of a veteran school psychologist. For example, a special education teacher in the program might be writing a behavior intervention plan for one of her students. The hours writing this plan may count as practicum hours, but only if this student collaborates with their school psychology practicum supervisor to finalize the intervention plan. In this situation, the GYO student is meeting her professional responsibilities while also accumulating practicum hours required for her graduate training. Ensuring students can meet responsibilities to both their employer and to the graduate program has been difficult. Requiring students to have these conversations with their employers immediately upon acceptance to the program has been helpful. Further, working with partner agencies to offer alternate employment options for program students wherein they have a more flexible schedule and easier access to school psychological supervision has also been successful. Similar supervision challenges arise in the DCT model, where accruing supervised clinical hours may require trainees to shift sites after internship to meet licensure requirements, potentially affecting district level retention. Applying similar strategies has been helpful, including requiring early discussions with internship sites about post internship supervision capacity, developing step down placement options that allow trainees to remain in region while accruing hours, and formalizing supervision agreements that clarify expectations and supervision access. Across both models, establishing sustainability structures that support continuous supervision through practicum, internship, and the supervised licensure period is essential for promoting trainee success and workforce retention.

Shifting roles for school psychologists in rural and other high-needs settings is something for which trainers must be continuously mindful. As we prepare school mental health providers for practice in these contexts, faculty should consider how to ensure trainees are equipped to meet the unique needs of students, educators, and families in communities where service systems are often stretched. Rural school psychologists report experiencing their work differently than their urban peers, including serving more buildings, traveling more miles, navigating dual relationships and confidentiality in small communities, and working within distinct cultural, religious, and political contexts (A. N. Goforth et al., 2017). Many of these challenges also characterize high-needs settings more broadly, where shortages, higher acuity, and competing demands can expand expected roles and compress time for prevention, consultation, and intensive intervention. Due to the dearth of mental health providers in rural communities—and similarly constrained access in many high-needs districts—school mental health providers are often called on to deliver a wider range of services than their urban peers, increasing the importance of strong training and ongoing professional learning to avoid practicing beyond the boundaries of one’s competence (Edwards & Sullivan, 2014; A. N. Goforth et al., 2017). Place-based training programs can address these contextual and ethical challenges explicitly during preparation, including instruction and coached practice related to rural and high-needs service delivery realities (e.g., cross-building workload management, consultation structures, confidentiality in small systems, and culturally responsive engagement), thereby better preparing school psychologists for practice (Skaar et al., in press).

To meet these demands, training programs can leverage complementary expertise across a supervisory and mentoring network that includes school psychology faculty, school psychologists who are dual-credentialed as fully licensed psychologists, and school psychologists who are dual-credentialed as independent mental and behavioral health providers. Faculty expertise can support strong grounding in NASP-aligned competencies, data-based decision making, and systems-level consultation, while dual-credentialed supervisors can provide practice-based mentorship that integrates educational and clinical standards, particularly when trainees are expected to provide intensive mental and behavioral health services in schools. This distributed expertise can strengthen trainees’ role clarity, ethical decision making, and scope-of-practice awareness, while also building capacity to deliver comprehensive, context-responsive services in rural and other high-needs schools (Edwards & Sullivan, 2014; A. N. Goforth et al., 2017; Skaar et al., in press).

NASP graduate standards are rigorous and are intended to ensure that school psychologists are well prepared across the 10 domains of practice. The most recent standards were adopted in 2020 just as the pandemic was beginning to erupt and university programs were forced to move online (National Association of School Psychologists [NASP], 2020b). Since that time, the number of online and hybrid training models has increased, and the use of flexible delivery formats to address school mental health workforce shortages has become a greater focus of professional associations and federal grant initiatives (National Association of School Psychologists [NASP], 2021; U.S. Department of Education, 2025). Programs like those described above must continue to adhere to rigorous national standards; however, accreditation agencies should consider greater flexibility in how programs demonstrate adherence to those standards, particularly as training pathways diversify and increasingly include respecialization and “grow your own” models for working professionals (National Association of School Psychologists [NASP], 2020c, 2021). In practice, many trainees entering these pathways bring more extensive applied skills than traditional students entering directly from undergraduate programs, and training programs must develop principled ways to honor relevant work experiences and previous graduate coursework while still ensuring competency-based mastery of required domains. This includes considering how classroom teaching, serving as a behavior interventionist in a rural or high-needs district, or completing related graduate coursework may reasonably count toward portions of training requirements, provided that the program can document that trainees meet NASP-aligned competencies (National Association of School Psychologists [NASP], 2020a, 2021).

Mental and behavioral health licensing agencies can further support this workforce development work by broadening or clarifying how they define acceptable graduate preparation and supervised training considering expanding scope across school and clinical settings, while maintaining rigorous standards for independent practice. In many states, licensure requirements were built with clinic-based training models in mind. As schools in rural states increasingly serve as a primary access point for mental and behavioral health care for children, licensure agencies can help reduce the burden on the workforce by recognizing high-quality, school-based clinical training experiences and by ensuring that supervision and competency requirements are clearly compatible with service delivery in multi-tiered educational settings (Frey et al., 2022; U.S. Department of Education, 2025). Coordination of credentialing and licensure expectations across educational and health sectors, such as aligning definitions of supervised practice, acceptable training settings, and competency-based documentation, could reduce barriers for trainees pursuing dual roles, improve workforce mobility, and strengthen pipelines into rural and high-needs schools, all while preserving the rigor needed to protect students and families (Frey et al., 2022; National Association of School Psychologists [NASP], 2021; U.S. Department of Education, 2025). For example, licensing agencies could explicitly recognize school-based clinical placements as acceptable training sites, permit structured tele-supervision in rural regions, adopt competency-based documentation portfolios, allow dual-supervision arrangements, and clarify how prior graduate coursework and relevant school-based clinical experiences may satisfy defined requirements when equivalency and competency are demonstrated.

### 4.2. Future Directions

Both models are described using small samples and early-stage cohorts, which restricts generalizability and limits causal inference about program effects. Outcomes rely primarily on supervisor ratings and self-report data, and more objective measures could offer stronger conclusions. The rural model also reflects constrained demographic diversity consistent with local rural workforce patterns, reinforcing the need for equity-focused recruitment strategies that broaden representation and strengthen culturally responsive practice capacity (Espinoza et al., 2018). The outcomes reported in this study focus primarily on supervisor-rated competency at the end of training, progression toward licensure, and short-term employment placement in rural and high-needs school settings. These indicators provide important evidence of program feasibility, trainee readiness for entry-level practice, and early workforce impact; however, they represent a limited subset of potential outcomes relevant to comprehensive evaluation of school-based mental health (SBMH) training models. In particular, the current data do not capture trainee competency growth over time, trainee satisfaction or perceived preparedness, long-term retention beyond the first few years of practice, or downstream effects on service delivery capacity within partner districts. Future evaluation efforts should prioritize longitudinal and multi-level outcome measurement to better understand the broader impact of these training models.

Future evaluation efforts should prioritize longitudinal and multi-level outcome measurement to better understand the broader impact of these training models. At the trainee level, this includes tracking growth in NASP-aligned competencies across training milestones, assessing trainee perceptions of preparedness and role clarity, and examining retention and career trajectories over time—outcomes that are increasingly emphasized in competency-based and implementation-informed workforce development frameworks (Forman et al., 2021; National Association of School Psychologists [NASP], 2021). At the systems level, future work should examine service delivery outcomes within partner districts, such as changes in access to school-based mental health services, caseload capacity, and continuity of care, as well as potential impacts on student access to prevention and intervention supports in high-needs schools. Incorporating these additional indicators would strengthen understanding of how training investments translate into sustained workforce capacity and improved access to mental health services for students, while also supporting more rigorous comparisons across training models and contexts.

Future iterations should employ training strategies that strengthen workforce through hybrid and online instructional modalities. The present iteration of the GYO program requires students to have a master’s degree to enter the program; however, there has been interest in expanding the program to students who have a bachelor’s degree. This would mean offering the complete Ed.S. program through hybrid and online instructional modalities. National Association of School Psychologists (National Association of School Psychologists [NASP], 2020b) graduate standard 1.1 requires school psychology graduate programs “to provide multiple and systematic opportunities through coursework, supervised practices, and other comprehensive program activities for candidates to develop and encourage an affiliation with peers, faculty, and the profession.” Opportunities for distance students and professionals to develop their affiliation with the school psychology community would need to be thoughtful and intentional (Ball et al., 2022).

Future expansion efforts must be evaluated in alignment of policy guidance, accreditation standards, and state regulatory frameworks governing both educator preparation and mental and behavioral health licensure. Expanding the GYO program to include candidates entering at the bachelor’s level aligns with federal workforce development priorities that emphasize early pipeline development, accessible preparation pathways, and place-based recruitment in high-needs and rural communities (U.S. Department of Education, 2025). Expansion would require instructional redesign, increased program length, and careful alignment with the National Association of School Psychologists’ (NASP) graduate training standards to ensure competency-based preparation and eligibility for credentialing (National Association of School Psychologists [NASP], 2020b, 2021). Bachelor’s-level entry pathways would require additional coursework in foundational psychology, research methods, and assessment principles, along with expanded practicum and internship placement capacity and supervisory infrastructure. Given persistent shortages of credentialed school psychologists available to provide field supervision in rural and high-needs districts (Clopton & Knesting, 2006; National Association of School Psychologists [NASP], 2023), policy support would be necessary to incentivize supervision roles, expand paid residency or apprenticeship models, and ensure sustainable funding for training expansion. Absent these supports, rapid scaling could strain institutional and district capacity, underscoring the need for phased implementation consistent with accreditation and workforce guidance (National Association of School Psychologists [NASP], 2020b).

Similarly, the use of telesupervision to support Dual-Credentialing Clinical Training (DCT) trainees across geographically dispersed regions aligns with recent federal and state policy discussions emphasizing flexibility in supervision models to address workforce shortages, particularly in rural areas (National Governors Association, 2024; U.S. Department of Education, 2025). While NASP’s supervision standards permit synchronous remote supervision when conducted in compliance with ethical and professional guidelines (National Association of School Psychologists [NASP], 2020b), requirements for supervised clinical practice toward independent mental health licensure remain governed by state-specific statutes and administrative rules that vary in their acceptance of telesupervision (Frey et al., 2022). As such, broader implementation of telesupervision would require coordination with state departments of health and human services, licensure boards, and educator credentialing agencies to clarify acceptable supervision modalities, documentation standards, and supervisor qualifications. Policy actions that explicitly recognize high-quality school-based clinical placements, permit structured tele-supervision in shortage regions, and support cross-sector supervision agreements could substantially improve feasibility while maintaining safeguards for ethical practice and service quality (Edwards & Sullivan, 2014; Frey et al., 2022). Future expansion should therefore prioritize pilot testing within existing regulatory frameworks, engagement with licensing authorities, and systematic evaluation of supervision quality and trainee outcomes, consistent with federal guidance promoting evidence-informed workforce development strategies in school-based mental health systems (U.S. Department of Education, 2025).

Collectively, these considerations suggest that sustainable expansion of school-based mental health training programs depends not only on program design, but also on coordinated policy action across education, licensure, and workforce systems to support supervision capacity, flexible credential pathways, and equitable access to training in high-needs contexts.

Expanding the programs may lead to increased enrollment and increased burden on field placement supervision. Given the already low numbers of school psychologists in rural areas, supervision of clinical training needs also to be considered. The present iteration of the DCT program requires for trainees to re-locate for in-person supervision and competency training; however, there has been discussion of establishing a tele-supervision model to support the DCT program model implementation across larger distances. One solution would be to utilize the state-wide school psychology workforce to support supervision of school psychologists in rural and high-needs areas through online, synchronous interactions. If the program continues to succeed in retaining rural and high-needs school psychologists, this problem of supervision would lessen over time.

## 5. Conclusions

School systems continue to face significant shortages in the SBMH workforce, reinforcing the need for scalable training pathways that increase access to comprehensive services while remaining responsive to local context. The two models described here demonstrate complementary strategies for addressing these workforce gaps by training school psychologists through targeted, evidence-informed approaches. The rural GYO model emphasizes place-based respecialization and workforce stabilization through partnership-driven recruitment, cohort-based support, strong mentoring and supervision, and meaningful financial incentives, while acknowledging the real logistical challenges of supervision and fieldwork in rural shortage contexts. The dual-credentialing model emphasizes expanding clinical capacity in schools by integrating school psychology preparation with supervised clinical training and licensure-aligned competencies, strengthening workforce flexibility to meet complex student needs within a multi-tiered system of service delivery as utilized in school-based settings.

Together, these models suggest that a two-pathway approach may be especially valuable because it addresses both sides of the workforce problem by addressing the number of individuals available to serve in high-need rural communities and the range of services school psychologists are prepared and authorized to deliver within schools. One pathway strengthens geographic retention through community-based recruitment and placement, while the other strengthens clinical scope and cross-system flexibility across diverse educational and mental and behavioral health contexts. The findings support continued investment in partnerships, incentives, and supervision infrastructures, as well as policies that allow accessible training and flexible credential pathways aligned with local workforce realities. Moving forward, scaling these models alongside rigorous, outcomes-focused evaluation has the potential to strengthen comprehensive school mental health systems and improve access to prevention and intervention supports for students with mental, behavioral, and academic needs.

## Figures and Tables

**Table 1:** Alignment of NASP Domains with Training Activities Across Programs.

NASP Domain of Practice	Grow Your Own (GYO): Example Training Activities	Dual-Credentialing Clinical Training (DCT): Example Training Activities
Domain 1 Data-Based Decision Making & Accountability	Conducting psychoeducational evaluations within trainees’ local districts; interpreting assessment data for eligibility and intervention planning under supervision of a school psychologist	Integrating psychoeducational assessment with clinical diagnostic formulations; using data to guide treatment planning and progress monitoring
Domain 2 Consultation & Collaboration	School-based consultation with teachers, administrators, and special education teams; participation in problem-solving teams and IEP meetings	Interdisciplinary consultation with educators, administrators, families, and mental health providers; coordinated care planning across systems
Domain 3 Academic Interventions & Instructional Supports	Designing and monitoring academic interventions aligned with MTSS frameworks; coaching educators on instructional supports	Supporting academic skills and executive functioning through Tier 2/Tier 3 intervention planning informed by clinical and educational data
Domain 4 Mental and Behavioral Health Services & Interventions	Delivering counseling and behavioral interventions in schools under supervision; applying evidence-based interventions to address for student emotional and behavioral needs	Delivering Tier 2 and Tier 3 clinical services (e.g., individual and group therapy), including documentation and supervision aligned with licensure standards
Domain 5 School-Wide Practices to Promote Learning	Participation in PBIS and MTSS leadership teams; supporting universal prevention efforts in rural schools	Supporting universal mental health promotion initiatives and advising schools on implementation of comprehensive SBMH frameworks
Domain 6 Services to Promote Safe and Supportive Schools	Crisis response participation, threat assessment team involvement, and school safety planning	Crisis intervention, risk assessment, and trauma-informed response delivered within clinical and school-based protocols
Domain 7 Family, School, and Community Collaboration	Engaging families in assessment and intervention processes; leveraging local community resources in rural contexts	Coordinating care with families and community mental health providers; facilitating referrals and continuity of services
Domain 8 Equitable Practices for Diverse Populations	Providing culturally responsive assessment and intervention in rural and high-needs schools; addressing barriers related to poverty and access	Applying culturally responsive clinical practices; addressing systemic inequities in access to care through school-based service delivery
Domain 9 Research & Evidence-Based Practice	Applying evidence-based interventions within school practice; program evaluation coursework and supervised application	Using evidence-based clinical interventions; supervision focused on fidelity, outcomes, and clinical decision making
Domain 10 Legal, Ethical, and Professional Practice	Training in special education law, ethical decision making, and professional role boundaries within school systems; especially targeting ethical dilemmas specific to rural school psychological practice.	Training in ethical and legal standards across education and mental health systems, including privacy and licensure-level documentation and supervision

**Table 2:** GYO Program of Study.

	**Summer 1**	
EDPSYCH 6240	Introduction to School Psychology	3
	**Semester 1 (Fall)**	
MEASRES 6283	Academic Assessment and Interventions	4
MEASRES 6270	Program Evaluation	3
	**Semester 2 (Spring)**	
MEASRES 6282	Individual Intellectual Assessment	4
	**Summer 2 (Session 1 & 2)**	
SPED 6260	Special Education Law and Policy	3
MEASRES 6284	Psychosocial Assessment	4
	**Semester 3 (Fall)**	
EDPSYCH 6270	Behavioral Interventions in School Settings	3
EDPSYCH 6290	Practicum III	2
	**Semester 4 (Spring)**	
EDPSYCH 6272	Systems Level Consultation	3
EDPSYCH 6290	Practicum IV	3
	**Summer 3 (Session 1 & 2)**	
MEASRES 6287	Early Childhood Assessment & Intervention	3
EDPSYCH 6260	Counseling Intervention in School	3
	**Semester 5 & 6 (Fall, Spring)**	
EDPSYCH 6291	Internship in School Psychology	6
	**Total Credits**	44

**Table 3:** Internship Evaluation Data for GYO Students.

NASP Domain	1	2	3	4	5	6	7	8	9	10
GYO Student 1	7.6	9.1	7.3	7.9	8.0	9.0	8.8	9.4	6.7	8.3
GYO Student 2	7.8	8.0	7.8	7.7	8.3	7.8	7.6	7.8	7.7	8.9
GYO Student 3	10.0	9.7	10.0	10.0	10.0	10.0	10.0	10.0	10.0	10.0
GYO Student 4	7.0	8.4	6.4	6.7	6.5	6.5	8.2	8.2	5.0	9.2
GYO Student 5	6.1	6.9	6.4	7.9	6.8	7.3	8.5	9.0	6.0	7.3
GYO Student 6	8.8	8.9	8.6	9.4	9.3	8.3	8.4	10.0	9.0	9.8
GYO Student 7	6.6	8.4	6.0	6.4	7.3	8.0	5.5	7.4	5.7	8.8
GYO Student 8	6.3	9.0	8.4	8.0	9.5	8.5	7.2	9.4	7.0	8.5
GYO Student 9	8.4	9.0	8.6	8.5	9.8	9.0	9.0	8.8	9.0	9.6
AVERAGE	7.6	8.6	7.7	8.1	8.4	8.3	8.1	8.9	7.3	8.9

**Table 4:** DCT Didactic Training Topics.

Week	
**Pre-Week (Early July)**	Orientation to medical and clinical systems Basic Life Support Assaultive Management Training Epic electronic health record orientation
**Week 1: Welcome & Foundations**	Program orientation Wellbeing and self-care Foundations of Comprehensive School Mental Health Clinical Heuristics Overview of Intellectual and Developmental Disabilities Psychology Department Integration
**Week 2: Comprehensive School-Based Mental Health (SBMH) Services**	Intensive behavioral consultation Assessment and consultation Comprehensive Clinical Services Nebraska Community Context
**Week 3: National SBMH Best Practices & Implementation**	Asynchronous completion of mental health promotion modules and cultural inclusivity modules Parent management Telehealth training
**Week 4: Preparing for Internship Activities**	Intensive clinical skills Internship preparation Site readiness activities Structured supervision preparation
**Early August (Transition to Placement)**	Group supervision launch Autism evaluation overview Autism Diagnostic Observation System (ADOS) training
**Ongoing (Throughout Internship Year)**	Group Supervision Site Specific Professional Development MTSS consultation Clinical Topics

**Table 5:** Licensure and Certification Outcomes for DCT Trainees.

Outcome	N	Percentage
Provisional mental health license at end of internship year (N = 5)	4	80
Continuing toward provisional mental health licensure (N = 5)	3	60
Achieved independent mental health licensure (N = 3)	2	67
Certified as school psychologists (state or national; N = 5)	4	80

**Table 6:** Post-Internship Placement to Complete Licensure Hours (N = 5).

Placement Pathway	N	Percentage
Moved to new site to complete independent licensure hours	4	80
Remained at DCT program site for two additional years to complete licensure	1	20

## Data Availability

The original contributions presented in this study are included in the article. Further inquiries can be directed to the corresponding author.
